# Net reclassification index in comparison of prognostic value of disseminated intravascular coagulation diagnostic criteria by Japanese Society on Thrombosis and Hemostasis and International Society on Thrombosis and Haemostasis: a multicenter prospective cohort study

**DOI:** 10.1186/s12959-023-00523-1

**Published:** 2023-08-07

**Authors:** Hirotaka Mori, Kayo Harada-Shirado, Noriaki Kawano, Mineji Hayakawa, Yoshinobu Seki, Toshimasa Uchiyama, Kazuma Yamakawa, Hiroyasu Ishikura, Yuhei Irie, Kenji Nishio, Noritaka Yada, Kohji Okamoto, Takayuki Ikezoe

**Affiliations:** 1 Department of Hematology, Fukushima Medical University, 1 Hikarigaoka, Fukushima, Fukushima, 960-1295 Fukushima Japan; 2 Department of Hematology, Miyazaki Prefectural Miyazaki Hospital, 5-30 Kita Takamatsu- machi, Miyazaki, 880-8510 Miyazaki Japan; 3 Department of Emergency Medicine, Hokkaido University Hospital, N14W5, Kita-ku, Sapporo, 060-8648 Hokkaido Japan; 4 Department of Hematology, Uonuma Institute of Community Medicine, Niigata University Medical and Dental Hospital, Urasa, Minamiuonuma-shi, Niigata, 4132, 949-7302 Japan; 5 Department of Laboratory Medicine, National Hospital Organization Takasaki General Medical Center, 36 Takamatsu-cho, Takasaki, 370-0829 Gunma Japan; 6 Department of Emergency and Critical Care Medicine, Osaka Medical and Pharmaceutical University, 2-7 Daigaku-machi, Takatsuki, 569-8686 Osaka Japan; 7 Department of Emergency and Critical Care Medicine, Faculty of Medicine, Fukuoka University, 7-45-1 Nanakuma Jonan-ku, Fukuoka, 814-0180 Fukuoka Japan; 8 Department of General Medicine, Nara Medical University, 840 Shijo-cho, Kashihara, 634-8522 Nara Japan; 9 Department of Surgery, Kitakyushu City Yahata Hospital, 2-6-2 Ogura Yahatahigashi-ku, Kitakyushu, 805-8534 Fukuoka Japan

**Keywords:** Disseminated intravascular coagulation, Diagnosis criteria, Prognosis, Mortality, Net reclassification improvement

## Abstract

**Background:**

We compared the prognostic value of the Japanese Society on Thrombosis and Hemostasis (JSTH) disseminated intravascular coagulation (DIC) diagnostic criteria with that of the International Society on Thrombosis and Haemostasis (ISTH) DIC diagnostic criteria for 28-day in-hospital mortality.

**Methods:**

We conducted a multicenter prospective cohort study involving two hematology departments, four emergency departments, and one general medicine department in Japan between August 2017 and July 2021. We assessed three ISTH DIC diagnostic criteria categories using low cutoff levels of D-dimer (low D-dimer), high cutoff levels of D-dimer (high D-dimer), and fibrinogen/fibrin degradation products (FDP) as fibrin-related markers. The main outcome was diagnosis-based category additive net reclassification index (NRI).

**Results:**

A total of 222 patients were included: 82 with hematopoietic disorders, 86 with infections, and 54 with other diseases. The 28-day in-hospital mortality rate was 14% (n = 31). The DIC rates diagnosed by the JSTH, ISTH-low D-dimer, high D-dimer, and FDP DIC diagnosis were 52.7%, 47.3%, 42.8%, and 27.0%, respectively. The overall category additive NRI by JSTH DIC diagnosis vs. ISTH-low D-dimer, high D-dimer, and FDP DIC diagnosis were − 10 (95% confidence interval [CI]: −28 to 8, *p* = 0.282), − 7.8 (95% CI: −26 to 10, *p* = 0.401), and − 11 (95% CI: −26 to 3, *p* = 0.131), respectively.

**Conclusions:**

JSTH criterion showed the highest sensitivity for DIC diagnosis that did not improve but reflected the same prognostic value for mortality evaluated using ISTH DIC diagnosis criteria. This finding may help clinicians to use JSTH DIC criterion as an early intervention strategy in patients with coagulopathy.

**Supplementary Information:**

The online version contains supplementary material available at 10.1186/s12959-023-00523-1.

## Background

Disseminated intravascular coagulation (DIC) is systemic hypercoagulation caused by various underlying diseases, including systemic infections, solid and hematological malignancies, and other conditions [1]. As there are no reference standard for DIC, some DIC scoring systems have been developed to evaluate the severity of coagulation for specific purposes. For example, the International Society on Thrombosis and Haemostasis (ISTH) DIC scores are widely used because these include global coagulation tests such as platelet counts, D-dimer, fibrinogen/fibrin degradation products (FDP), and prothrombin time or prothrombin time-international normalized ratio (PT-INR), which can be measured in local laboratories and are generally employed in routine care [1]. In contrast, the Japanese Society on Thrombosis and Hemostasis (JSTH) DIC scores are composed of advanced and sensitive molecular coagulation markers, including antithrombin (AT), thrombin-antithrombin complex (TAT), soluble fibrin (SF), and prothrombin fragment F1 + 2 (F1 + 2), in addition to global coagulation tests [2].

DIC scoring systems are also useful for predicting poor outcomes. A multicenter observational validation study of 1,895 patients showed that patients with ISTH DIC had higher in-hospital mortality than those without DIC (38% vs. 24%, *p* < 0.001) [3]. Similarly, the JSTH DIC scoring systems were correlated with the odds ratio for mortality in patients with various underlying diseases, such as hematopoietic disorders, infection, and others [4–6].

This clinical background raises the question of how clinicians should use different diagnostic criteria to predict patient prognosis because no study has investigated how better other diagnostic criteria reclassify the prognosis of patients evaluated by certain criteria. Accordingly, this prospective cohort study directly assessed the prognostic value of the JSTH and ISTH DIC criteria for mortality. We employed a statistical method called the category net reclassification index (NRI) to compare the prognostic abilities of the two models [7].

## Materials and methods

### Study design and setting

This multicenter prospective cohort study used coagulopathy data (planned a priori) from the Japanese Society on Thrombosis and Hemostasis (JSTH) committee registry. This study was registered with the University Hospital Medical Information Network Clinical Trial Registry in August 2017 (UMIN-CTR ID: UMIN000032972). Data were collected from August 2017 to July 2021 from two hematology centers (Fukushima Medical University Hospital and Miyazaki Prefectural Miyazaki Hospital), four emergency centers (Hokkaido University Hospital, Nara Medical University Hospital, Osaka General Medical Center, and Fukuoka University Hospital), and one clinical laboratory department in an acute hospital (Takasaki General Medical Center) in Japan for a total of seven centers. All patients or their families provided written informed consent approved by the ethics committee of each institution before collecting patient data and blood samples. Our statistical analysis followed the Standards for Reporting Diagnostic Accuracy (STARD) statement [8] presented in the Supplementary Table S1 (Supplementary Material 1). The STARD statement was used since our study was related to medical tests, and the statement explains that most STARD items would still apply to evaluation of prognosis.

### Patients

We consecutively included patients with coagulopathy according to the following inclusion criteria: (1) age ≥ 16 years, (2) requiring hospitalization or urgent care for the treatment of underlying diseases [2], in addition to (3) laboratory coagulopathy data; platelet ≤ 120 × 10^3^/µL, fibrinogen ≤ 150 mg/dL, or FDP ≥ 10 µg/mL, or (4) patients evaluated by their physicians as meeting these laboratory coagulopathy data if the underlying disease is untreated. The exclusion criteria were as follows: (1) coagulation disorders due to obstetric and gynecological diseases; or (2) blood transfusion performed before the assessment of the inclusion criteria. We collected sample and data of coagulation tests at the time of urgent hospitalization or the initiation of treatment for underlying diseases.

### Data collection

We developed a clinical research form and collected the following data: age, sex, underlying diseases, and underlying disease types using the JSTH classification, including hematopoietic disorders, infectious diseases, and others classified as basic diseases [2]; laboratory tests including platelet counts, D-dimer, FDP, PT-INR, fibrinogen, AT, TAT, SF, F1 + 2, liver failure, administration of recombinant human soluble thrombomodulin (rhTM), administration of antithrombin, and 28-day in-hospital mortality. The choice of anticoagulation therapy was at the discretion of each physician. Samples for laboratory tests were collected prior to treatment. The underlying diseases of all the patients were treated according to the attending physician’s decisions. Platelet count, PT-INR, and fibrinogen levels were analyzed using an automated counting device at each institution. Plasma samples were stored at − 80 °C after centrifugation and sent to the assay companies for other coagulation tests. Latex photometric immunoassay measured D-dimer, FDP and SF levels, synthetic substrate assays measured AT levels, chemiluminescent enzyme immunoassay measured TAT levels at the LSI Medience Corporation (Tokyo, Japan), and enzyme-linked immunosorbent assay measured F1 + 2 levels at the Siemens Healthcare Diagnostics (Marburg, Germany).

DIC was diagnosed according to the JSTH diagnostic criteria [9] (supplementary Table S2; Supplementary Material 2) and ISTH diagnostic criteria [10] (supplementary Table S3; Supplementary Material 3). When the ISTH DIC diagnostic criteria were used, we used D-dimer and FDP (ISTH-FDP) as fibrin-related markers. In addition, we investigated cases where points of D-dimer were stratified according to low cut-off levels (from 0.4 µg/mL to less than 4.0 µg/mL, 2 points; over 4.0 µg/mL, 3 points) (ISTH-low-D-dimer) and high cut-off levels (from 3 µg/mL to less than 7 µg/mL, 2 points; over 7 µg/mL, 3 points) (ISTH-high D-dimer).

The attending physician chose which anticoagulant, antithrombin, or DIC scoring systems were used. Compliance with the data form was also monitored. For missing information, we interviewed physicians who completed the data form. We conducted these post hoc interviews within three months of obtaining data from the laboratory companies.

### Outcome measurement

The main outcome was 28-day in-hospital mortality at the time of study entry. Patients discharged after treatment completion or those who remained in the hospital for > 28 days were considered alive. We confirmed the survival of the patients who were transferred to other departments within 28 days.

### Statistical analysis

First, we summarized patients’ characteristics using medians and interquartile ranges for continuous variables and percentages for categorical variables for survivors and non-survivors. Continuous and categorical variables were compared between survivors and non-survivors using Mann–Whitney–Wilcoxon or chi-square tests, respectively.

Second, we evaluated the association between mortality and DIC diagnostic criteria for both JSTH and ISTH. We calculated the diagnostic rate, mortality rate, odds ratios (ORs), area under the receiver operating characteristic curve (AUC), sensitivity and specificity of the diagnostic criteria of JSTH and ISTH.

Third, to compare the predictive ability of the JSTH DIC diagnostic criteria and the ISTH DIC diagnostic criteria for mortality, we calculated a diagnosis-based category additive NRI [7]. The category NRI assesses how much better a new model is at evaluating the risk categories compared with a previous model. The additive NRI was calculated by adding the percentage of patients with an event correctly reclassified (No. with an event having a higher risk in model A than in model B – No. with the event that had a lower risk in model A than in model B/total No. of patients with the event × 100) to the percentage of patients without the event correctly reclassified (No. without an event having a lower risk in model A than in model B – No. without an event having a higher risk in model A than in model B/total No. of patients without an event × 100). Generally, NRI evaluates the reclassification by a new model A, developed with the aim of improving the existing model B. The additive NRI can range from 200 (all patients with the event had greater risk, and all patients without the event had a lower risk in model A than in model B) to − 200 (the opposite). In this study, the event was defined as 28-day in-hospital mortality. High- or low-risk was defined as the diagnosis of DIC. Model A corresponds to the JSTH DIC criteria and model B corresponds to each ISTH DIC criterion. The *p*-value in the NRI is based on the hypothesis that the JSTH DIC criteria do not improve the reclassification of patients according to the ISTH DIC criteria.

A 95% confidence interval (95% CI) was calculated, and statistical significance was set at *p*-value < 0.05. A complete case analysis was then performed. Therefore, we did not estimate the sample size a priori and used all available samples. Data were analyzed using STATA software, V. 15 (StataCorp., College Station, TX, USA) and R software, V.4.1.2 (http://www.r-project.org).

## Results

### Patient characteristics

Table 1 presents patient characteristics. A total of 222 patients from seven hospitals were eligible for this study. Among these patients, 31 (14%) died. There were no statistically significant differences in age; percentage of males or females; percentage of underlying diseases; levels of FDP, D-dimer, SF, and F1 + 2; and percentage of liver failure between survivors and non-survivors. There were statistically significant differences in platelet counts, PT-INR, fibrinogen levels, AT, and TAT and percentage of administration of rhTM between survivors and non-survivors. There was one patient with missing TAT values and 17 patients with missing values of F1 + 2.

**Table 1:** Patient characteristics

	Survivor	Non-survivor		*p*-value	
	n = 191	n = 31			
Demographics					
Age (years)	68 (56 to 78)	71 (60 to 79)		0.34	
Male	115 (60.2)	19 (61.3)		0.91	
Underlying disease types				0.25	
Hematopoietic disorders	71 (64.5)	11 (35.5)			
Infectious diseases	77 (71.0)	9 (29.0)			
The others	43 (64.5)	11 (35.5)			
Laboratory tests					
Platelets (× 10^3^/µL)	69 (40 to 164)	39 (14 to 128)		0.006	
FDP (µg/mL)	22.05 (11.5 to 44.7)	22.72 (13.7 to 116.7)		0.19	
D-dimer (µg/mL)	14.6 (8.5 to 28.0)	13.0 (8.5 to 70.0)		0.68	
PT-INR	1.2 (1.05 to 1.41)	1.4 (1.2 to 1.54)		0.003	
Fibrinogen (mg/dL)	270 (186 to 422)	212 (128 to 282)		0.013	
AT (%)	85.8 (67.8 to 101.9)	74.8 (59.3 to 88.5)		0.013	
TAT* (ng/mL)	10.85 (6.09 to 30.7)	32.18 (11 to 145.73)		< 0.001	
SF (µg/mL)	43.4 (18.1 to 103)	52.9 (17.5 to 215)		0.23	
F1 + 2† (pmol/L)	748 (416 to 1201)	849.5 (324 to1201)		0.99	
Liver failure	NA	1 (3.2)		NA	
Anti-DIC agents					
rhTM	81 (42.6)	21 (67.7)		0.009	
Antithrombin	39 (20.5)	11 (35.5)		0.065	

FDP, fibrinogen/fibrin degradation products; PT-INR, prothrombin time-international normalized ratio; fibrinogen, antithrombin; AT, thrombin-antithrombin complex; TAT, soluble fibrin; SF, prothrombin fragment F1 + 2, F1 + 2; rhTM, recombinant human soluble thrombomodulin; NA, not applicable

* One patient had a missing TAT value

† There were 17 patients with missing F1 + 2

Data on demographics, underlying disease types, liver failure, and anti-DIC agents are presented as n (%). Laboratory test results are presented as median (interquartile range).

### Diagnosis rate, mortality rate and prognostic values of JSTH and ISTH DIC

Table 2 shows the DIC diagnosis and 28-day mortality rates of the JSTH and ISTH diagnostic criteria. The DIC diagnosis rate by JSTH, ISTH-low D-dimer, high D-dimer, and FDP diagnosis criteria were 52.7% (n = 117), 47.3% (n = 105), 42.8% (n = 95), and 27.0% (n = 60), respectively. The 28-day mortality rates of JSTH-DIC, ISTH-low D-dimer, high D-dimer, and FDP-DIC were 18.0% (21/117), 21.0% (22/105), 21.1% (20/95), and 26.7% (16/60), respectively. The ORs of JSTH DIC, ISTH-low D-dimer, high D-dimer, and FDP DIC for mortality were 2.08 (95% CI: 0.93 to 4.65, *p* = 0.075), 3.18 (95% CI: 1.39 to 7.27, *p* = 0.006), 2.81 (95% CI: 1.28 to 6.20, *p* = 0.010), and 3.56 (95% CI: 1.63 to 7.78, *p* = 0.001), respectively. The AUC of JSTH, ISTH-low D-dimer, high D-dimer, and FDP DIC diagnostic criteria were 0.59 (95%CI: 0.50 to 0.68), 0.64 (95%CI: 0.55 to 0.73), 0.63 (95%CI: 0.53 to 0.72), and 0.64 (0.55 to 0.74), respectively (supplementary Fig. S1; Supplementary Material 4). There was no difference in the AUC (*p* = 0.41). The sensitivity and specificity of JSTH, ISTH-low D-dimer, high D-dimer, and FDP DIC diagnostic criteria were 67.7% and 49.7%, 71.0% and 56.6%, 64.5% and 60.7%, 51.6% and 77.0%, respectively. Supplementary Table S4 (Supplementary Material 5) shows the accordance and discordance of diagnosis by JSTH diagnostic criteria vs. each ISTH diagnostic criteria. The accordance rate of diagnosis for DIC positive and negative between JSTH DIC diagnosis vs. ISTH-low D-dimer, high D-dimer, and FDP DIC diagnosis were 36.5% and 36.5%, 39.2% and 34.7%, and 47.3% and 27.0%, respectively. The discordance of diagnosis between positive and negative JSTH DIC diagnosis vs. negative and positive ISTH-low D-dimer, high D-dimer, and FDP DIC diagnosis were 16.2% and 10.8%, 18.1% and 8.1%, and 25.7% and 0%, respectively.

**Table 2:** Diagnosis rate and 28-day mortality rates of JSTH DIC and ISTH DIC

Diagnostic criteria	DIC Diagnosis	Survivors n = 191	Non-survivors n = 31
JSTH			
DIC +	117 (52.7)	96 (82.0)	21 (18.0)
DIC −	105 (47.3)	95 (90.5)	10 (9.5)
ISTH-low D-dimer*			
DIC +	105 (47.3)	83 (79.0)	22 (21.0)
DIC −	117 (52.7)	108 (92.3)	9 (7.7)
ISTH-high D-dimer†			
DIC +	95 (42.8)	75 (78.9)	20 (21.1)
DIC −	127 (57.2)	116 (90.5)	11 (9.5)
ISTH-FDP‡			
DIC +	60 (27.0)	44 (73.3)	16 (26.7)
DIC −	162 (73.0)	147 (89.7)	15 (9.3)

DIC, disseminated intravascular coagulation; JSTH, Japanese Society on Thrombosis and Hemostasis; ISTH, International Society on Thrombosis and Haemostasis

* ISTH-low D-dimer used a low cut-off level of D-dimer as a fibrin-related marker

† ISTH-high D-dimer used a high cut-off level of D-dimer as a fibrin-related marker

‡ ISTH-FDP uses FDP as a fibrin-related marker

Data are presented as n (%). In the DIC diagnosis row, the proportion of DIC positive or negative patients among all patients is presented as a percentage. In the row of survivors and non-survivors, the proportion of the number of survivors or non-survivors among DIC positive or negative patients is presented as percentages.

### NRI by JSTH DIC vs. ISTH DIC

Tables 3 and 4, and 5 shows the diagnosis-based reclassification of the JSTH DIC criteria versus each ISTH DIC criterion.

**Table 3:** Reclassification table of JSTH DIC criteria vs. ISTH-low D-dimer DIC criteria

ISTH-low D-dimer*	JSTH	
	DIC -	DIC +
In 191 survivors		
DIC -	75	33
DIC +	20	63
In 31 non-survivors		
DIC -	6	3
DIC +	4	18

NRI = (20 − 33) × 100/191 + (3 − 4) × 100/31 = − 10 (95%CI: − 28 to 8), *p*-value = 0.282

DIC, disseminated intravascular coagulation; JSTH, Japanese Society on Thrombosis and Hemostasis; ISTH, International Society on Thrombosis and Haemostasis; NRI, net reclassification index

* ISTH-low D-dimer used a low cut-off level of D-dimer as a fibrin-related marker.

**Table 4:** Reclassification table of JSTH DIC criteria vs. ISTH-high D-dimer DIC criteria

ISTH-high D-dimer*	JSTH	
	DIC -	DIC +
In 191 survivors		
DIC -	80	36
DIC +	15	60
In 31 non-survivors		
DIC -	7	4
DIC +	3	17

NRI = (15 − 36) × 100/191 + (4 − 3) × 100/31 = − 7.8 (95%CI: − 26 to 10), *p*-value = 0.401

DIC, disseminated intravascular coagulation; JSTH, Japanese Society on Thrombosis and Hemostasis; ISTH, International Society on Thrombosis and Haemostasis; NRI, net reclassification index

* ISTH-high D-dimer used a high D-dimer cut-off level as a fibrin-related marker

**Table 5:** Reclassification table of JSTH DIC criteria vs. ISTH-FDP DIC criteria

ISTH-FDP*	JSTH	
	DIC -	DIC +
In 191 survivors		
DIC -	95	52
DIC +	0	44
In 31 non-survivors		
DIC -	10	5
DIC +	0	16

NRI = (0 − 52) × 100/191 + (5 − 0) × 100/31 = − 11 (95%CI: −26 to 3), *p*-value = 0.131

DIC, disseminated intravascular coagulation; JSTH, Japanese Society on Thrombosis and Hemostasis; ISTH, International Society on Thrombosis and Haemostasis; NRI, net reclassification index

* ISTH-FDP uses FDP as a fibrin-related marker

The overall category additive NRI by JSTH DIC diagnosis criteria vs. ISTH-low D-dimer, high D-dimer, and FDP DIC diagnosis criteria were − 10 (95% CI: −28 to 8, *p* = 0.282), − 7.8 (95% CI: −26 to 10, *p* = 0.401), and − 11 (95% CI: −26 to 3, *p* = 0.131), respectively. The net reclassification numbers by JSTH DIC diagnosis criteria vs. ISTH-low D-dimer, high D-dimer, and FDP DIC diagnosis criteria in survivors and non-survivors were − 13 (− 6.8%) and − 1 (− 3.2%), − 21 (− 11.0%) and 1 (3.2%), and − 52 (− 27.2%) and 5 (16.1%), respectively. Supplementary Table S5 (Supplementary Material 6) shows the NRI separated by type of underlying disease. We examined the risk of bias of administration of rhTM since the Table 1 found the statistical significance of rhTM administration between survivors and non-survivors. In survivors and non-survivors, there was no bias in the administration of rhTM between patients with JSTH DIC and non-ISTH-low D-dimer and high D-dimer DIC and patients with ISTH-low D-dimer and high D-dimer DIC and non-JSTH DIC (Supplementary Table S6; Supplementary Material 7 and Supplementary Table S7; Supplementary Material 8). There were no patients with ISTH-FDP DIC and non-JSTH DIC (Supplementary Table S8; Supplementary Material 9) and the comparison of the administration of rhTM between these patients was not applicable.

## Discussion

This multicenter prospective cohort study included 222 patients with coagulopathy caused by various underlying diseases, the distributions of which were well balanced. The overall mortality rate was 14% (31/222). This study compared the prognostic value of the JSTH DIC diagnostic criteria and the three ISTH DIC diagnostic criteria for mortality. The DIC diagnosis rate was the highest in the JSTH DIC criteria (52.7%) and the lowest in the ISTH-FDP DIC criteria (27.0%). In contrast, the 28-day mortality rate of JSTH DIC was the lowest (18.0%) and that of ISTH-FDP was the highest (26.7%). This proportion reflected the lowest ORs for JSTH DIC (2.08, 95% CI:0.93 to 4.65) and the highest ORs for ISTH-FDP DIC (3.56, 95% CI:1.63 to 7.78). There was no statistical difference in AUC of JSTH and ISTH diagnostic criteria for mortality.

This study is the first report of NRI to compare the prognostic value of JSTH and ISTH DIC in the frame of clinical usefulness. The results showed that the JSTH diagnostic criteria did not better reclassify the prognosis of the patients evaluated using the ISTH DIC diagnosis criteria. The NRI showed negative reclassification, especially in survivors, although the difference was not statistically significant. In addition, we judged that the net reclassification number in non-survivors according to the JSTH DIC diagnostic criteria was too low to place more clinical value on the classification of non-survivors than on survivors. These results indicate that the JSTH DIC diagnostic criteria tended to over-evaluate coagulopathy compared to the ISTH DIC diagnostic criteria. Because recent cohort studies indicated that the identification of high mortality risk patients with coagulopathy was an efficient strategy to maximize the effect of anticoagulants and to improve the outcome [11, 12], these oversensitive JSTH criteria may not contribute significantly to improving the treatment strategy for DIC. However, some overestimation by JSTH DIC criteria may be accepted as an early anticoagulation strategy because a limitation of a randomized clinical trial was the delay in administration of rhTM, which might decrease the benefit of the anticoagulation [13]. Therefore, a further randomized clinical trial comparing the prognostic value of JSTH and other DIC diagnostic criteria with an indication of anticoagulant administration should be conducted.

We also investigated the causes of reclassification by JSTH diagnostic criteria vs. each ISTH diagnostic criteria. Around 10% of JSTH DIC negative patients were diagnosed as DIC by ISTH low and high cutoff D-dimer criteria. On the other hands, all of JSTH DIC negative patients showed negative DIC by ISTH-FDP criteria. The accordance and discordance of diagnosis was caused by D-dimer and FDP. Because JSTH diagnosis criteria adapts FDP as fibrin related marker, the accordance rate of DIC negative between JSTH and ISTH-FDP diagnostic criteria was higher than the accordance rate between JSTH and ISTH- D-dimer diagnostic criteria. In addition, the specificity of ISTH-low and high D-dimer cutoff levels for mortality were lower than that of ISTH-FDP diagnostic criteria. As a previous review pointed out, the interpretation of D-dimer levels, which are influenced by a variety of factors, necessitates the clinician’s expertise, the clinical setting, and other available laboratory analyses [14]. Collectability, these findings suggests that some of DIC patients by ISTH-D-dimer criteria may be the status of pseudo-positive DIC although there is no gold standard criteria of DIC.

This study had some limitations. First, we did not have a sufficient sample size to evaluate the prognostic value of JSTH DIC scores because the criteria of the JSTH DIC scoring system vary among underlying disease types [9], and the scores cannot be compared among patients with different underlying disease types. In this study, the patients’ background showed statistical differences in AT and TAT, which suggests that JSTH DIC scores may be useful to assess the severity of coagulation, although the median TAT levels in survivors and non-survivors met the criteria for scoring points. Therefore, further statistical methods, such as category-free NRI, integrated discrimination improvement, and decision curve analysis, should be used to evaluate the predictive value of JSTH DIC scores [15]. Due to the small sample size, the analysis of the prognosis of JSTH DIC criteria separated by the underlying disease types may have a risk of beta error and be optimistic. Second, we did not evaluate the prognostic value of the Japanese Ministry of Health and Welfare (JMHW) [16], and Japanese Association for Acute Medicine (JAAM) criteria [17]. Our registry lacked a plan to collect JMHW and JAAM score data from the same patient, and the available data were sparse. Third, the 18% mortality rate of JSTH DIC patients was lower than the 35% mortality rate in a registry study [18] probably because this study was conducted in tertiary referral centers offering intensive care. This low mortality rate may contribute to the worse reclassification of survivors according to the JSTH DIC criteria. Fourth, information on other anticoagulants, such as heparin or serine protease inhibitors, was not evaluated. This limitation decreased the generalizability of our findings in other countries where rhTM is not used as an anticoagulant. To warrant generalizability, a prospective and multicenter study including patients with various diseases and risk factors should be conducted [19].

## Conclusions

JSTH DIC diagnostic criterion did not improve the prognostic value for mortality evaluated by the ISTH DIC diagnostic criteria. However, the JSTH DIC diagnostic criterion showed the highest sensitivity to diagnose DIC, indicating that JSTH criterion may be useful for early diagnosis of DIC. ISTH D-dimer-criterion may include pseudo positive DIC based on JSTH criterion, and ISTH-FDP criterion reflected the highest specificity and odds ratio for mortality. These findings suggest that FDP may be suitable as a fibrin related marker. Overall, the prognostic value of JSTH and ISTH DIC diagnostic criteria remains low and further improvement is needed.

### Electronic supplementary material

Below is the link to the electronic supplementary material.

Supplementary Material 1

Supplementary Material 2

Supplementary Material 3

Supplementary Material 4: Supplementary fig S1. AUC of JSTH and ISTH DIC diagnostic criteria for mortality. AUC, area under the receiver operating characteristic curve, DIC, disseminated intravascular coagulation; JSTH, Japanese Society on Thrombosis and Hemostasis; ISTH, International Society on Thrombosis and Haemostasis. ISTH-low D-dimer used a low cut-off level of D-dimer as a fibrin-related marker. ISTH-high D-dimer used a high cut-off level of D-dimer as a fibrin-related marker. ISTH-FDP uses FDP as a fibrin-related marker.

Supplementary Material 5

Supplementary Material 6

Supplementary Material 7

Supplementary Material 8

Supplementary Material 9

## Data Availability

Data are available upon reasonable request to the corresponding author.

## Abbreviations

DIC: disseminated intravascular coagulation
ISTH: International Society on Thrombosis and Haemostasis
JSTH: Japanese Society on Thrombosis and Haemostasis
STARD: The Standards for Reporting Diagnostic Accuracy
FDP: fibrinogen/fibrin degradation products
PT-INR: prothrombin time international normalized ratio
AT: antithrombin
TAT: thrombin-antithrombin complex
SF: soluble fibrin
F1 + 2: prothrombin fragment F1 + 2
rhTM: recombinant human soluble thrombomodulin
ORs: odd ratios
AUC: area under the operating characteristic curve
NRI: net reclassification index
