# Supplementary material for: Net reclassification index in comparison of prognostic value of disseminated intravascular coagulation diagnostic criteria by Japanese Society on Thrombosis and Hemostasis and International Society on Thrombosis and Haemostasis: a multicenter prospective cohort study
Source: Thromb J. 2023 Aug 7;21:84. doi: 10.1186/s12959-023-00523-1 (PMC10405497; doi:10.1186/s12959-023-00523-1)
Supplement: Supplementary file 2 — Supplementary Material 2 [file 12959_2023_523_MOESM2_ESM.docx]

| **Supplementary Table S2. JSTH DIC scoring system *** | | | |
| --- | --- | --- | --- |
| **Items** | **Hematopoietic disorders** | **Infectious** | **Basic** |
| Platelet counts (× 10^3^/µL)  　> 120  　> 80 to 120  　> 50 to 80  ≤ 50 | NA | 0  1  2  3 | 0  1  2  3 |
| FDP（μg/mL）  < 10  10 to < 20  20 to < 40  ≥ 40 | 0  1  2  3 | 0  1  2  3 | 0  1  2  3 |
| Fibrinogen（mg/dL）  　> 150  　> 100 to 150  ≤ 100 | 0  1  2 | NA | 0  1  2 |
| PT-INR  　< 1.25  　1.25 to < 1.67  　≥ 1.67 | 0  1  2 | 0  1  2 | 0  1  2 |
| Antithrombin (%)  ≥ 70  < 70 | 0  1 | 0  1 | 0  1 |
| TAT, SF, or F1+2  < 2-fold of normal upper limit  ≥ 2-fold of normal upper limit† | 0  1 | 0  1 | 0  1 |
| Liver failure  No  YES | 0  −3 | 0  −3 | 0  −3 |
| DIC diagnosis | ≥ 4 | ≥ 5 | ≥ 6 |

* Wada H, et al. DIC subcommittee of the Japanese Society on Thrombosis and Hemostasis. The revised diagnostic criteria for DIC have been approved by the Japanese Society for Thrombosis and Hemostasis. Thromb J. 2017 3; 15:17.

† TAT ≥ 6 ng/mL, 1 point; SF ≥ 14 μg/mL, 1point; F1+2 ≥ 460 pmol/L, 1point.
