# Supplementary material for: Net reclassification index in comparison of prognostic value of disseminated intravascular coagulation diagnostic criteria by Japanese Society on Thrombosis and Hemostasis and International Society on Thrombosis and Haemostasis: a multicenter prospective cohort study
Source: Thromb J. 2023 Aug 7;21:84. doi: 10.1186/s12959-023-00523-1 (PMC10405497; doi:10.1186/s12959-023-00523-1)
Supplement: Supplementary file 3 — Supplementary Material 3 [file 12959_2023_523_MOESM3_ESM.docx]

| **Supplementary Table S3. ISTH DIC scoring system *** | | | |
| --- | --- | --- | --- |
| **Items** | **Low D-dimer**† | **High D-dimer**‡ | **FDP§** |
| Platelet counts (× 10^3^/µL)  　≥ 100  　50 to < 100  　< 50 | 0  1  2 | 0  1  2 | 0  1  2 |
| D-dimer（μg/mL）  　< 0.4  　0.4 to < 4.0  　≥ 4.0 | 0  2  3 | NA | NA |
| D-dimer（μg/mL）  　< 3  　3 to < 7  　≥ 7 | NA | 0  2  3 | NA |
| FDP（μg/mL）  　< 10  　10 to < 40  　≥ 40 | NA | NA | 0  2  3 |
| PT-INR  　< 1.4  　1.4 to < 2.3  　≥ 2.3 | 0  1  2 | 0  1  2 | 0  1  2 |
| Fibrinogen（mg/dL）  　≥ 100  　< 100 | 0  1 | 0  1 | 0  1 |
| DIC diagnosis | ≥ 5 | ≥ 5 | ≥ 5 |

* Suzuki K, et al. Subcommittee on Disseminated Intravascular Coagulation. A re-evaluation of the D-dimer cut-off value for making a diagnosis according to the ISTH overt-DIC diagnostic criteria: Communication from the SSC of the ISTH. J Thromb Haemost. 2018; 16:1442-1444.

† Low D-dimer levels indicate low cutoff levels of D-dimer used as fibrin-related markers.

‡ High D-dimer levels indicate high cut-off levels of D-dimer used as fibrin-related markers.

**§** ISTH-FDP uses FDP as a fibrin-related marker.
