# Supplementary figures and images for: Net reclassification index in comparison of prognostic value of disseminated intravascular coagulation diagnostic criteria by Japanese Society on Thrombosis and Hemostasis and International Society on Thrombosis and Haemostasis: a multicenter prospective cohort study
Source: Thromb J. 2023 Aug 7;21:84. doi: 10.1186/s12959-023-00523-1 (PMC10405497; doi:10.1186/s12959-023-00523-1)

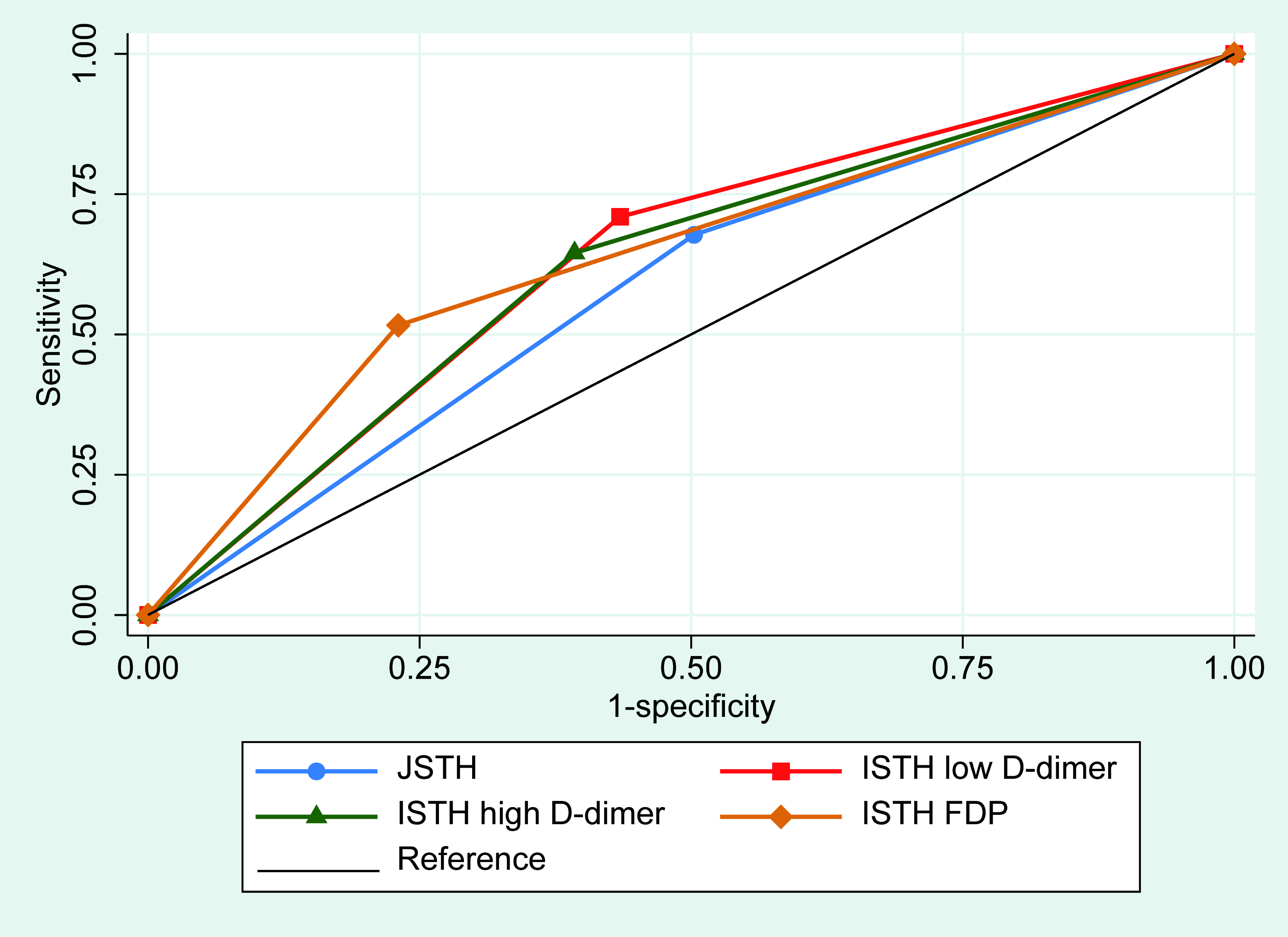

Supplement: Supplementary file 4 — Supplementary Material 4: Supplementary fig S1. AUC of JSTH and ISTH DIC diagnostic criteria for mortality. AUC, area under the receiver operating characteristic curve, DIC, disseminated intravascular coagulation; JSTH, Japanese Society on Thrombosis and Hemostasis; ISTH, International Society on Thrombosis and Haemostasis. ISTH-low D-dimer used a low cut-off level of D-dimer as a fibrin-related marker. ISTH-high D-dimer used a high cut-off level of D-dimer as a fibrin-related marker. ISTH-FDP uses FDP as a fibrin-related marker. [file 12959_2023_523_MOESM4_ESM.tif]
