# Supplementary material for: Net reclassification index in comparison of prognostic value of disseminated intravascular coagulation diagnostic criteria by Japanese Society on Thrombosis and Hemostasis and International Society on Thrombosis and Haemostasis: a multicenter prospective cohort study
Source: Thromb J. 2023 Aug 7;21:84. doi: 10.1186/s12959-023-00523-1 (PMC10405497; doi:10.1186/s12959-023-00523-1)
Supplement: Supplementary file 5 — Supplementary Material 5 [file 12959_2023_523_MOESM5_ESM.docx]

| **Supplementary Table S4. Accordance and discordance of JSTH DIC criteria vs. ISTH DIC criteria in all patients** | | |
| --- | --- | --- |
| **ISTH** | **JSTH** | |
|  | **DIC -** | **DIC +** |
| low D-dimer* |  |  |
| DIC - | 81 (36.5%) | 36 (16.2%) |
| DIC + | 24 (10.8%) | 81 (36.5%) |
|  |  |  |
| high D-dimer† |  |  |
| DIC - | 87 (39.2%) | 40 (18.0%) |
| DIC + | 18 (8.1%) | 77 (34.7) |
|  |  |  |
| FDP‡ |  |  |
| DIC - | 105 (47.3%) | 57 (25.7%) |
| DIC + | 0 (0%) | 60 (27.0%) |

DIC, disseminated intravascular coagulation; JSTH, Japanese Society on Thrombosis and Hemostasis; ISTH, International Society on Thrombosis and Haemostasis

* ISTH-low D-dimer used a low cut-off level of D-dimer as a fibrin-related marker.

† ISTH-high D-dimer used a high cut-off level of D-dimer as a fibrin-related marker.

‡ ISTH-FDP uses FDP as a fibrin-related marker.
