# Supplementary material for: Net reclassification index in comparison of prognostic value of disseminated intravascular coagulation diagnostic criteria by Japanese Society on Thrombosis and Hemostasis and International Society on Thrombosis and Haemostasis: a multicenter prospective cohort study
Source: Thromb J. 2023 Aug 7;21:84. doi: 10.1186/s12959-023-00523-1 (PMC10405497; doi:10.1186/s12959-023-00523-1)
Supplement: Supplementary file 6 — Supplementary Material 6 [file 12959_2023_523_MOESM6_ESM.docx]

| **Supplementary Table S5. Reclassification table of JSTH DIC criteria vs. ISTH DIC criteria in patients with hematopoietic disorders, infectious, and basic types** | | | | | | |
| --- | --- | --- | --- | --- | --- | --- |
|  | **JSTH** | | | | | |
| **ISTH** | **Hematopoietic** | | **Infectious** | | **Basic** | |
|  | **DIC -** | **DIC +** | **DIC -** | **DIC +** | **DIC -** | **DIC +** |
| Low D-dimer* |  |  |  |  |  |  |
| No. of survivors | 71 |  | 77 |  | 43 |  |
| DIC - | 25 | 8 | 26 | 15 | 24 | 10 |
| DIC + | 16 | 22 | 2 | 34 | 2 | 7 |
| No. of non-survivors | 11 |  | 9 |  | 11 |  |
| DIC - | 0 | 0 | 2 | 2 | 4 | 1 |
| DIC + | 4 | 7 | 0 | 5 | 0 | 6 |
| NRI | −25 (−57 to 6)  *p*-value = 0.17 | | 5 (−24 to 34)  *p*-value = 0.72 | | −9.5 (−32 to 13)  *p*-value = 0.41 | |
|  |  | |  | |  | |
| High D-dimer† |  |  |  |  |  |  |
| No. of survivors | 71 |  | 77 |  | 43 |  |
| DIC - | 29 | 9 | 27 | 17 | 24 | 10 |
| DIC + | 12 | 21 | 1 | 32 | 2 | 7 |
| No. of non-survivors | 11 |  | 9 |  | 11 |  |
| DIC - | 1 | 0 | 2 | 2 | 4 | 2 |
| DIC + | 3 | 7 | 0 | 5 | 0 | 5 |
| NRI | −23 (−52 to 6)  *p*-value = 0.12 | | 1 (−27 to 30)  *p*-value = 0.92 | | 0.4 (−28 to 27)  *p*-value = 0.98 | |
|  |  | |  | |  | |
| FDP‡ |  |  |  |  |  |  |
| No. of survivors | 71 |  | 77 |  | 43 |  |
| DIC - | 41 | 12 | 28 | 28 | 26 | 12 |
| DIC + | 0 | 18 | 0 | 21 | 0 | 5 |
| No. of non-survivors | 11 |  | 9 |  | 11 |  |
| DIC - | 4 | 1 | 2 | 2 | 4 | 2 |
| DIC + | 0 | 6 | 0 | 5 | 0 | 5 |
| NRI | −7.8 (−27 to 11)  *p*-value = 0.42 | | −14 (−43 to 15)  *p*-value = 0.34 | | −9.7 (−36 to 17)  *p*-value = 0.47 | |

DIC, disseminated intravascular coagulation; JSTH, Japanese Society on Thrombosis and Hemostasis; ISTH, International Society on Thrombosis and Haemostasis; NRI, net reclassification index.

* ISTH-low D-dimer used a low cut-off level of D-dimer as a fibrin-related marker.

† ISTH-high D-dimer used a high cut-off level of D-dimer as a fibrin-related marker.

‡ ISTH-FDP uses FDP as a fibrin-related marker.
