# Supplementary material for: Net reclassification index in comparison of prognostic value of disseminated intravascular coagulation diagnostic criteria by Japanese Society on Thrombosis and Hemostasis and International Society on Thrombosis and Haemostasis: a multicenter prospective cohort study
Source: Thromb J. 2023 Aug 7;21:84. doi: 10.1186/s12959-023-00523-1 (PMC10405497; doi:10.1186/s12959-023-00523-1)
Supplement: Supplementary file 7 — Supplementary Material 7 [file 12959_2023_523_MOESM7_ESM.docx]

| **Supplementary Table S6. Comparison of administration of** **recombinant human soluble thrombomodulin between JSTH DIC criteria and ISTH-low D-dimer DIC criteria** | | |
| --- | --- | --- |
| **ISTH-low D-dimer*** | **JSTH** | |
|  | **DIC -** | **DIC +** |
| In 191 survivors |  |  |
| DIC - | 16/75 (21.3) | 15/33 (45.5) |
| DIC + | 9/20 (45.0) | 41/63 (65.1) |
| In 31 non-survivors |  |  |
| DIC - | 1/6 (16.7) | 2/3 (66.7) |
| DIC + | 3/4 (75.0) | 15/18 (83.3) |

DIC, disseminated intravascular coagulation; JSTH, Japanese Society on Thrombosis and Hemostasis; ISTH, International Society on Thrombosis and Haemostasis.

Data are presented as n (%).

There was no difference in the administration rate of recombinant human soluble thrombomodulin between patients with JSTH DIC and non-ISTH DIC and patients with ISTH DIC and non-JSTH DIC among survivors (*p* = 0.97) and non-survivors (*p* = 1.0).

Data are presented as n (%).

* ISTH-low D-dimer used a low cut-off level of D-dimer as a fibrin-related marker.
