# Supplementary material for: Net reclassification index in comparison of prognostic value of disseminated intravascular coagulation diagnostic criteria by Japanese Society on Thrombosis and Hemostasis and International Society on Thrombosis and Haemostasis: a multicenter prospective cohort study
Source: Thromb J. 2023 Aug 7;21:84. doi: 10.1186/s12959-023-00523-1 (PMC10405497; doi:10.1186/s12959-023-00523-1)
Supplement: Supplementary file 8 — Supplementary Material 8 [file 12959_2023_523_MOESM8_ESM.docx]

| **Supplementary Table S7. Comparison of administration of recombinant human soluble thrombomodulin between JSTH DIC criteria and ISTH-high D-dimer DIC criteria** | | |
| --- | --- | --- |
| **ISTH-high D-dimer*** | **JSTH** | |
|  | **DIC -** | **DIC +** |
| In 191 survivors |  |  |
| DIC - | 17/80 (21.3) | 15/36 (41.7) |
| DIC + | 8/15 (53.3) | 41/60 (68.3) |
| In 31 non-survivors |  |  |
| DIC - | 2/7 (28.6) | 3/4 (75.0) |
| DIC + | 2/3 (66.7) | 14/17 (82.4) |

DIC, disseminated intravascular coagulation; JSTH, Japanese Society on Thrombosis and Hemostasis; ISTH, International Society on Thrombosis and Haemostasis.

Data are presented as n (%).

There was no difference in the administration rate of recombinant human soluble thrombomodulin between patients with JSTH DIC and non-ISTH DIC and patients with ISTH DIC and non-JSTH DIC among survivors (*p*-value = 0.446) and non-survivors (*p*-value = 1.0).

* ISTH-high D-dimer used a high D-dimer cut-off level as a fibrin-related marker.
