# Supplementary material for: Net reclassification index in comparison of prognostic value of disseminated intravascular coagulation diagnostic criteria by Japanese Society on Thrombosis and Hemostasis and International Society on Thrombosis and Haemostasis: a multicenter prospective cohort study
Source: Thromb J. 2023 Aug 7;21:84. doi: 10.1186/s12959-023-00523-1 (PMC10405497; doi:10.1186/s12959-023-00523-1)
Supplement: Supplementary file 9 — Supplementary Material 9 [file 12959_2023_523_MOESM9_ESM.docx]

| **Supplementary Table S8. Comparison of administration of recombinant human soluble thrombomodulin between JSTH DIC criteria and ISTH-FDP DIC criteria** | | |
| --- | --- | --- |
| **ISTH-FDP*** | **JSTH** | |
|  | **DIC -** | **DIC +** |
| In 191 survivors |  |  |
| DIC - | 25/95 (26.3) | 26/52 (50.0) |
| DIC + | 0 (NA) | 30/44 (68.2) |
| In 31 non-survivors |  |  |
| DIC - | 4/10 (40.0) | 4/5 (80.0) |
| DIC + | 0 (NA) | 13/16 (81.3) |

DIC, disseminated intravascular coagulation; JSTH, Japanese Society on Thrombosis and Hemostasis; ISTH, International Society on Thrombosis and Hemostasis; NA, not applicable.

Data are presented as n (%).

We were not able to compare the proportion of administration of recombinant human soluble thrombomodulin between patients with JSTH DIC and non-ISTH DIC and between patients with ISTH DIC and non-JSTH DIC in survivors and non-survivors.

Data are presented as n (%).

* ISTH-FDP uses FDP as a fibrin-related marker.
